# Domestic and wild animal samples and diagnostic testing for SARS-CoV-2

**DOI:** 10.1080/01652176.2023.2263864

**Published:** 2023-10-02

**Authors:** Megan R. Miller, Elias Braun, Hon S. Ip, Gregory H. Tyson

**Affiliations:** a Center for Veterinary Medicine, U.S. Food and Drug Administration, Laurel, MD, USA; b School of Veterinary Medicine, University of PA, Philadelphia, PA, USA; c National Wildlife Health Center, U.S. Geological Survey, Madison, WI, USA

**Keywords:** SARS-CoV-2, diagnostics, PCR, variant, ELISA, NGS, VNT

## Abstract

From the first cases in 2019, COVID-19 infections caused by severe acute respiratory syndrome coronavirus 2 (SARS-CoV-2) have resulted in over 6 million human deaths in a worldwide pandemic. SARS-CoV-2 is commonly spread from human to human through close contact and is capable of infecting both humans and animals. Worldwide, there have been over 675 animal outbreaks reported that resulted in over 2000 animal infections including domestic and wild animals. As the role of animal infections in the transmission, pathogenesis, and evolution of SARS-CoV-2 is still unfolding, accurate and reliable animal diagnostic tests are critical to aid in managing both human and animal health. This review highlights key animal samples and the three main diagnostic approaches used for animal testing: PCR, serology, and Next Generation Sequencing. Diagnostic results help inform (often difficult) clinical decision-making, but also possible ways to mitigate spread among pets, food supplies, or wildlife. A One Health approach has been key to monitoring the SARS-CoV-2 pandemic, as consistent human-animal interactions can lead to novel variants. Having multiple animal diagnostic tests for SARS-CoV-2 available is critical to ensure human, animal, and environmental health.

## 1. Introduction

Severe acute respiratory syndrome coronavirus 2 (SARS-CoV-2) emerged in 2019 from Wuhan, China and has resulted in over 612 million human cases and 6.53 million deaths (CDC). Coronaviruses (CoVs) are a group of enveloped viruses with a positive single-stranded RNA genome and are organized into four genera: α, β, γ, and δ-coronaviruses (Naqvi et al. 2020). A characteristic of coronavirus is a relatively fast mutation rate; the estimated substitution rate of SARS-CoV-2 is 1.3 × 10-3 per-base per-infection cycle (Amicone et al. 2022). The SARS-CoV-2 genome is 32 kb in length with a 5′-cap, a 3′-poly-A tail, four structural proteins, spike (S), envelope (E), membrane (M) and nucleocapsid (N) and 16 non-structural proteins. A frameshift between ORF1a and ORF1b results in the production of two polypeptides that are processed into the non-structural proteins (Mariano et al. 2020). The S protein is critical to binding to the host cell receptor, angiotensin I converting enzyme 2 (ACE2), through the S1 subunit that contains the receptor-binding domain (RBD) (Scialo et al. 2020). ACE2 is conserved among mammals which allows for SARS-CoV-2 to have a wide host range. SARS-CoV-2 can be transmitted through zoonotic transmission and is spread human to human (Hayashi et al. 2020). Of concern, are situations where humans and animals in close contact, e.g. companion animals/owners, production animals/workers, zoo animals/workers, and as exemplified by the situation with white-tailed deer, perhaps even hunters and wildlife professionals with wildlife (Chan et al. 2022).

Reverse zoonotic spread of diseases in domestic, production, wildlife, and zoological animals can lead to direct impact on the animals involved, including potential outbreaks. These impacts can even blend into federal or state regulatory actions such as removal of products or culling of animals. For this reason, it is crucial that diagnostic tests are accurate and can be performed as rapidly as possible to ensure that appropriate responses can be quickly initiated. In the United States, many organizations performing animal diagnostics consult guidelines set forth by the American Association of Veterinary Laboratory Diagnosticians (AAVLD) (Toohey-Kurth et al. 2020). To date, animal SARS-CoV-2 infections span over 36 countries across the Americas, Africa, Asia, and Europe, with 29 different animal species affected during 675 documented outbreaks (FAO 2023). A wide range of cross-species transmission often leads to species-specific adaptations and these evolutionary changes can impact pathogenicity, transmissibility, and vaccine efficacy.

As the role of animal infections on the transmission and pathogenesis of SARS-CoV-2 is still unfolding, accurate and reliable animal diagnostic tests are critical. Specificity and sensitivity are critical parameters to any diagnostic test. It is important to recognize that many assays were developed in the absence of recognized standards, especially when granted emergency use authorization (EUA) by federal authorities. Sensitivity of an assay can also be measured by the limit of detection (LOD), that is, the lowest detectable number of viral copies that resulted in a positive at least 95% of the time. Another way specificity is also commonly determined is by testing the assay with a panel of pathogens commonly found in the same sample type. Importantly, as diagnostic tests are developed for new species or SARS-CoV-2 variants, specificity and sensitivity should be re-evaluated. Here we will discuss samples used for diagnostic tests and the three main diagnostics tests used in veterinary medicine: PCR-based, serology and NGS.

## 2. Animal samples for diagnostic testing

A range of animal samples can be used for testing; the most widely used for PCR and NGS-based tests are nasal swabs or oropharyngeal swabs; fecal, urine, serological, and post-mortem tissue samples can also be used (Klaus et al. 2021). The breadth of sampling sites and strategies are important considerations given the range of species and possible samples. SARS-CoV-2 infects the respiratory tract of many animals, although there are organ tropism differences among variants. Assay results can vary for multiple reasons with tissue type, tissue handling, and storage as considerations.

### 2.1. Respiratory and gastrointestinal samples

Early in the pandemic respiratory infection was well-documented in cats and mink along with gastrointestinal distress (Mahdy et al. 2020; Lenz et al. 2022). Infection in deer leads to shedding in their nasal mucus and feces, indicating respiratory and gastrointestinal infection, although little illness is reported (Chandler et al. 2021). Additionally, feces from deer had less viral RNA and infectious virus than oral and nasal swabs (Martins et al. 2022). Similarly, fecal samples from tigers had lower viral loads than samples from the respiratory tract (McAloose et al. 2020). Due to consistent infection of the respiratory tract and higher viral loads, nasal or oropharyngeal swabs are most reliable for detecting SARS-CoV-2 during active infection.

### 2.2. Tissue samples

SARS-CoV-2 has been documented to have tissue tropism to the kidney (Puelles et al. 2020; Caceres et al. 2021; Diao et al. 2021), which may lead to SARS-CoV-2 RNA and protein being shed in urine. Studies in animals have found that experimental infection in ferrets leads to detectable amounts of viral RNA in the urine up to 8 days post-infection (Mahdy et al. 2020). Tissues are also used to determine if the animal was infected with SARS-CoV-2 at the time of death and are collected postmortem. Experimental infections in macaques (Boszormenyi et al. 2021), dogs, and cats (Gaudreault et al. 2020; Rudd et al. 2021) showed that viral RNA can be detected in a wide range of tissues, including lymph nodes, nasal turbinate, trachea, spleen, liver, heart, and kidneys. Importantly, viral RNA was detected up to 21 days post infection in tissues, however viral loads were 4-log lower and detected less frequently than samples from the respiratory tract (Gaudreault et al. 2020). Experimental infections are inoculated with higher viral loads then those seen in natural infection. Therefore, tissues from natural infections are less reliable and have variation in detection of SARS-CoV-2. A case study that looked at ten naturally infected cats and dogs showed that virus is sparsely distributed in tissues, as samples from the same tissue gave differing results from multiple tests (virus isolation, RT-qPCR, histology, and pathology) (Carpenter et al. 2021). This indicates that detection from animal tissue is unreliable for diagnostics and should be substituted for more reliable sampling sites (oropharyngeal) when available. Of note, nasal swabs, oropharyngeal swabs, and fecal swabs can all be collected postmortem.

### 2.3. Sample handling and processing

Tissue handling and processing from the time from infection to collection and storage can affect virus detection (Beattie et al. 2022). Nasal swabs, oropharyngeal swabs, feces/fecal swabs, urine, and tissues can all have a matrix effect that can interfere with SARS-CoV-2 detection (Morais et al. 2022). Matrix effects are cause in part by enzymes and microbial, including yeast and fungi communities that can break down pathogens, this can alter detection. Animal diagnostic tests, especially tests adapted from human diagnostics, should account for, and reduce the impact of matrix effects. Until research is done to better identify and minimize matrix effects on animal origin samples, many follow guidelines set forth by the CDC for sample collection and storage in humans (CDC 2022). For animal samples, the USDA mirrors the CDC guidelines and recommend storing nasal, oropharyngeal, and fecal swabs in viral transport media or saline, depending on the assay, at 2-8 °C for up to 72 h. For testing beyond 72 h, it is recommended to store the sample or extracted RNA at −70 °C (USDA).

## 3. Polymerase chain reaction (PCR)

One of the most widely used pathogen detection methods for active infection in animals is PCR, which amplifies a targeted portion of a pathogen’s nucleic acids. Detection of active infection of SARS-CoV-2 can happen two to seven days post-infection (Sethuraman et al. 2020). For many RNA viruses, such as SARS-CoV-2, reverse-transcription quantitative PCR (RT-qPCR) is considered the ‘gold-standard’ diagnostic tool. In this protocol extracted RNA from diagnostic samples is converted into cDNA by the reverse transcriptase enzyme. The resulting cDNA product then undergoes PCR amplification with complementary primers and a fluorescent probe which enables relative real-time quantification of viral load (Zhang, Huang et al. 2022).

Current SARS-CoV-2 RT-qPCR assays target various parts of the viral genome including E, S, N, RBD, and the ORFs (Zhang, Huang et al. 2022). The most common SARS-CoV-2 animal assay is the 2019-nCoV CDC RT-qPCR assay that targets the N gene. Multiple other assays, with varying targets, have been designed under the EUA issued for human testing by the U.S. Food and Drug Administration (FDA n.da). These tests are not dependent on species, which has allowed for transition from human to animal samples for screening of veterinary and wildlife samples. In the U.S. to date the following species have been confirmed SARS-CoV-2 positive by PCR: cats, dogs, tigers, lions, gorillas, snow leopards, otters, hyenas, binturongs, coatis, cougars, fishing cats, lynx, mandrills, and squirrel monkeys (USDA 2023).

A comparison between 45 veterinary laboratories determined the rate of detection of SARS-CoV-2 in between different markers; most labs use N1 and N2 markers with a LOD of 20 copies or less. Additionally, there was a > 97% specificity and sensitivity amongst labs (Deng et al. 2021, 2022). When animal nasal matrix was accounted for, specificity decreased slightly (Deng, Nemser et al. 2022). Four common SARS-CoV-2 diagnostic assays, the RdRp-SARSr (Charite) assay targeting E and RdRp, HKU assay targeting N and nsp14, China CDC assay targeting N and nsp10, and the U.S. CDC assay targeting two N sections, were compared and showed that three of the assays can detect 500 RNA copies per reaction; the RdRp-SARSr assay was less sensitivity and had 6-10 higher CT values (Vogels et al. 2020). The US CDC 2019-nCoV assay, which is commonly used for human and animal diagnostics, is reported to have a LOD at one copy for N1 and five for N2 and was tested with 20 other pathogens for a 100% specificity (CDC 2021). Importantly, these indicate that overall PCR is very specific and sensitive regardless of extraction method, matrix or variant, however N gene targets are more reliable.

Variations of PCR have been developed to offer an ever-growing array of advantages. Multiplex PCR assays screen for numerous genetic targets within a single reaction (Akhtardanesh et al. 2023). Multiplex assays can be more time and cost effective through analyzing a single sample for different pathogens that could present similarly in the animal. As an example, a study using the CDC Influenza SARS-CoV-2 (Flu SC2) multiplex assay showed human to canine SARS-CoV-2 transmission during which both experienced respiratory signs (Wendling et al. 2022).

Nested PCR (nPCR) is the amplification of a small targeted genetic segment contained within the amplicon of a traditional PCR reaction using two separate pairs of primers. The nPCR process results in increased test sensitivity and specificity. Sirakov et al. developed a nPCR protocol for SARS-CoV-2 detection in cats with 100% sensitivity and specificity at low RNA concentrations (Sirakov et al. 2022). Nested PCR targeting the S protein was also used for detection of SARS-CoV-2 in deer with low viral loads so that substandard samples could be sequenced (Marques et al. 2022).

Unlike traditional PCR, Loop- mediated isothermal amplification (LAMP) is a methodology that does not require temperature cycling. LAMP, which has been used to detect SARS-CoV-2 in human samples, is advantageous due to its cost efficiency and minimal equipment needs (Huang, Tang et al. 2022). To date LAMP has not been used on animal samples for SARS-CoV-2 but has been previously used to detect Feline Coronavirus, highlighting the potential of this technology for animal diagnostics (Rapichai et al. 2022). Other nucleic acid amplification tests are being developed including digital droplet PCR (ddPCR) which was used in a case series to diagnose SARS-CoV-2 infection in cats and dogs with suspect myocarditis (Ferasin et al. 2021).

Overall RT-qPCR is one of the most sensitive assays to detect SARS-CoV-2 and is commonly used in diagnostic labs. Table 1 is a non-exhaustive list of the PCR assays referred to in this review and demonstrates the vast diversity of species tested, targets, and PCR methods. The relationship between RT-qPCR and antigen was evaluated and showed that antigen tests have a stronger correlation to virus recovery and that antigen tests have higher specificity and produce fewer false positives (Currie et al. 2022). However, RT-qPCR is more sensitive and produces fewer false negatives.

**Table 1.** Commercial and in-house PCRs for SARS-CoV-2 detection.

Assay	Reference	Species	Target	Method
CDC 2019-nCoV Panel	(Lenz et al. 2022; McAloose et al. 2020; Gaudreault et al. 2020; Ferasin et al. 2021; Jairak et al. 2022)	Dog, Cat, Tiger, Lion	N N	RT-qPCR ddPCR
Tetracore Inc.® EZ-SARS-CoV-2 assay (Tetracore, #TC-5048-192)	(Martins et al. 2022)	Deer	N	RT-qPCR
SENMURV Mulristar-2 SARS-COV-2 kit (Stem Cell Technology, Iran; # BONCOV-MS2200PB)	(Akhtardanesh et al. 2023)	Cat	N, RdRp	RT-qPCR
CDC Influenza SARS-CoV-2 (Flu SC2) Multiplex Assay	(Wendling et al. 2022)	Dog	N	Multiplex RT-qPCR
Cobas® 6800 SARS-CoV-2 Test (Roche)	(Hoppe et al. 2023)	Dog	N, E	Multiplex RT-qPCR
OPTI Medical SARS-CoV-2 kit (OPTI Medical System)	(Kuchipudi et al. 2022)	Deer	N	RT-qPCR
TaqPath™ COVID‑19 CE‑IVD Kit (Thermo Fisher Scientific, #A48067)	(Zoccola et al. 2021)	Cat	ORF1ab, N, S	Multiplex RT-qPCR
In house PCR	(Munnink et al. 2021; Chan et al. 2022; Goldberg et al. 2022; Arteaga et al. 2022; Roundy et al. 2022; McAloose et al. 2020; Boszormenyi et al. 2021; Marques et al. 2022; Jairak et al. 2022; Klaus et al. 2021; Fernandez-Bellon et al. 2021; Schiaffino et al. 2021; Rivero et al. 2022; Sanchez-Morales et al. 2022; Montagutelli et al. 2021)	Dog, Cat, Mink, Hamster, Opossum, Squirrel, Raccoon, Fox, Deer, Rabbit, Bobcat, Tiger, Lion, Macaques, Mice	RdRp, N, E, S	RT-qPCR
	(Arteaga et al. 2022)	Armadillo	RdRp	Pan-Corona nPCR
	(Sirakov et al. 2022)	Cat	N	nPCR
	(Ferasin et al. 2021)	Dog, Cat	S	ddPCR
	(Pickering et al. 2022)	Deer	UTR, E	Mutiplex RT-qPCR

N: nucleocapsid; RdRp: RNA-dependent RNA polymerase; E: envelope; S: Spike; UTR: untranslated region; RT-qPCR: reverse-transcription-quantitative PCR; nPCR: nested PCR; ddPCR: digital drop PCR.

## 4. Serology by enzyme-linked immunosorbent assay (ELISA) and virus neutralization test (VNT)

After a week of infection, humoral immune response develops and as the virus is cleared, PCR becomes less reliable as an indicator of disease status. The production of antibodies may last up to one year, but the titer decreases significantly after 6 months. Studies vary on the exact length of time that antibodies can be detected. One study showed antibody levels in naturally infected cats are only detected up to 100 days post infection (Zhang, Zhang et al. 2020), however, others indicate that antibodies in companion animals are stable and can be detected up to 10 months (Hamer et al. 2021, Decaro et al. 2022). Serological assays, such as ELISA, determine antibody titers in serum. Most ELISA detect IgG and are used to look for evidence of past infection. IgM is useful for determining infection timeline as IgM is observed early in infection. Household pets who were seropositive for IgM were also positive for IgG; however, not all IgG positive pets were seropositive for IgM, indicating some pets were more recently infected than others (Bienzle et al. 2022). IgG antibody tests were developed for several animal species as understanding infection in animals became more critical. ELISA assays detect antibodies within a sample and are most accurate when species specific. To date there are ELISAs optimized for cattle, swine, chicken, rabbit, and cats (Bold et al. 2022, Fritz et al. 2022; Gontu et al. 2022). An indirect multi-species ELISA based on the RBD was developed and tested on serum from ferrets, raccoon dogs, hamsters, rabbits, chickens, cattle and cats with an overall specificity of 100.0% and sensitivity of 98.31% (Wernike et al. 2021) (Table 2). There is a commercially available multi-species ELISA from Innovative Diagnostics (ID Screen® SARS-CoV-2 Double Antigen Multi-species ELISA) that has been used to test many species (Barua et al. 2021; Laidoudi et al. 2021; Davoust et al. 2022; Jairak et al. 2022) (Table 2). Currently in the U.S., SARS-CoV-2 antibodies have been confirmed in cats, dogs, and lions (USDA). Kainulainen et al. developed a high-throughput protein complementation assay to use as a serological assay multispecies, this assay detected antibodies in 92.5% of samples, with 100% specificity (Kainulainen et al. 2021). This assay was later used to determine antibodies in canines (Wendling et al. 2022).

**Table 2.** Commercial and in-house ELISA for SARS-CoV-2 antibody detection.

Assay	Reference	Species	Target	Method
CDC 2019-nCoV Panel	(Gaudreault et al. 2020; McAloose et al. 2020; Ferasin et al. 2021; Lenz et al. 2022; Jairak et al. 2022)	Dog, Cat, Tiger, Lion	N	RT-qPCR ddPCR
Tetracore Inc.® EZ-SARS-CoV-2 assay (Tetracore, #TC-5048-192)	(Martins et al. 2022)	Deer	N	RT-qPCR
SENMURV Mulristar-2 SARS-COV-2 kit (Stem Cell Technology, Iran; # BONCOV-MS2200PB)	(Akhtardanesh et al. 2023)	Cat	N, RdRp	RT-qPCR
CDC Influenza SARS-CoV-2 (Flu SC2) Multiplex Assay	(Wendling et al. 2022)	Dog	N	Multiplex RT-qPCR
Cobas® 6800 SARS-CoV-2 Test (Roche)	(Hoppe et al. 2023)	Dog	N, E	Multiplex RT-qPCR
OPTI Medical SARS-CoV-2 kit (OPTI Medical System)	(Kuchipudi et al. 2022)	Deer	N	RT-qPCR
TaqPath™ COVID‑19 CE‑IVD Kit (Thermo Fisher Scientific, #A48067)	(Zoccola et al. 2021)	Cat	ORF1ab, N, S	Multiplex RT-qPCR
In house PCR	(McAloose et al. 2020; Boszormenyi et al. 2021; Fernandez-Bellon et al. 2021; Klaus et al. 2021; Montagutelli et al. 2021; Munnink et al. 2021; Schiaffino et al. 2021; Arteaga et al. 2022; Chan et al. 2022; Goldberg et al. 2022; Marques et al. 2022; Jairak et al. 2022; Rivero et al. 2022; Roundy et al. 2022; Sanchez-Morales et al. 2022)	Dog, Cat, Mink, Hamster, Opossum, Squirrel, Raccoon, Fox, Deer, Rabbit, Bobcat, Tiger, Lion, Macaques, Mice	RdRp, N, E, S	RT-qPCR
	(Arteaga et al. 2022)	Armadillo	RdRp	Pan-Corona nPCR
	(Sirakov et al. 2022)	Cat	N	nPCR
	(Ferasin et al. 2021)	Dog, Cat	S	ddPCR
	(Pickering et al. 2022)	Deer	UTR, E	Mutiplex RT-qPCR

NC = not calculated, N = nucleocapsid, RBD = Receptor Binding Domain, S = spike.

VNT is considered the gold standard for detection of neutralizing SARS-CoV-2 antibody, mainly against S and RBD, in pets. VNTs are commonly used along with ELISA to validate results, however inconsistency between ELISA and VNTs often occurs due to lack of antibodies with neutralizing activities and difference in viral antigens used (Ratti et al. 2022). Serological assays aim to have 100% sensitivity and specificity, as limiting false positives and negatives are critical to diagnostics. VNTs use serum to determine if an animal has been previously infected with SARS-CoV-2. VNT assays measure the neutralizing antibodies in serum through *in vitro* virus neutralization. Importantly, VNT for SARS-CoV-2 are non-species-specific, but sensitivity depends on virus variant and require BSL-3 space. Several studies use VNTs to validate ELISA data and determine past infections, however cutoffs and stock virus variants differ between studies which lead to differences in results. Early in the pandemic most studies used the Wuhan 2020 variant, although isolated from different sources, NY (Martins et al. 2022), IL, (Roundy et al. 2022), WA (Barua et al. 2021; Bold et al. 2022; Gontu et al. 2022). Other studies used variants that emerged later on during the pandemic, such as GNL-1205 (isolated from a patient at UTMB on 7/6/2021) (Goldberg et al. 2022), 2 SARS-CoV-2 D6124G variant (B.1 lineage) and the Gamma variant (Arteaga et al. 2022), and EPI-ISL-418268, an isolate from a patient in Barcelona, Spain (Fernandez-Bellon et al. 2021). As antibodies in serum are specific to the variant of infection, the variant used in VNT may influence the neuralization and must be considered. RBD of the Wuhan Strain of SARS-CoV-2 as the antigen in ELISA showed a reduction in cross-neutralization on other variants including Beta, Gamma, and Omicron (Gontu et al. 2022).

An alternative method to VNTs that can be done at a BSL-2 level is the SARS-CoV-2 Surrogate Virus Neutralization Test (sVNT) from GenScript. This assay is used by many labs to detect antibodies in a range of species or as a validation to in-house ELISAs (Barua et al. 2021; Chandler et al. 2021; Fernandez-Bellon et al. 2021; Klaus et al. 2021; Hoppe et al. 2023; Jairak et al. 2022). For SARS-CoV-2 ELISA have overall given a higher rate of antibody detection than VNT, and there may be several factors that influence this. For one, ELISA will pick up all antibodies that bind to the target antigen not just those that neutralize. There is also the potential for cross-detection of other coronaviruses, which can lead to false positives. Second, VNTs use live virus and the variant used will affect detection. If the test serum has antibodies to the Wuhan variant and the VNT is using an Omicron variant this will potentially decrease detection. These are factors that must be considered when using ELISAs and VNTs for diagnostics. A comparison of two commercial ELISA kits and the surrogate virus neutralization test (sVNT) along with the conventional VNT demonstrates the variation of specificity and sensitivity among serological assays. VNT found 6.8% positive samples, the sVNT had 100% sensitivity, while the commercial ELISA kits had a sensitivity of 94–97%. Specificity was high amongst all assays >99% (Ratti et al. 2022). Overall, serological assays are important diagnostic tools however the specificity and sensitivity of each assay should be evaluated.

Cutoff values are also selected by the researcher and can be reported at an inhibition of 50% (Bold et al. 2022; Goldberg et al. 2022), 66.7% (Gontu et al. 2022), or 90% (Roundy et al. 2022). Antibody detection is critical to determine the rate of infection in a population and the potential risk of transmission, however acknowledging factors that influence results and including secondary validation methods like VNTs, is key. In one study, ELISA results were compared to VNT across three species: cattle, swine and chicken. Overall, there was a strong correlation between titers from ELISA and VNT, however, ELISAs gave slightly higher titers (Gontu et al. 2022). Another validation method is western blot, as western blot can be used to determine antibodies in serum. By ELISA 25 out of 44 dogs were positive for SARS-CoV-2 antibodies and 68% of those were also positive by western blot (Laidoudi et al. 2021). Another study using western blot to determine the presence of SARS-CoV-2 antibodies had varying reactivity against the five viral protein targets, with most samples only reacting to one or two targets (Davoust et al. 2022), indicating the importance and relevance of viral proteins. Another factor to consider is how researchers determine positivity when testing against multiple antigens, for example seroprevalence in rabbits was 0.7% using criteria of positive results from two antigens or 1.4% for only one antigen (Fritz et al. 2022).

## 5. Next generation sequencing (NGS)

Sequencing of SARS-CoV-2 suspect samples has been used to confirm diagnoses, identify variants and survey for genetic changes that might affect assay performance or alter biological function. Importantly, some sequencing platforms show >96% accuracy when samples have a CT value of 30 or lower (Malta et al. 2021) however, samples with CT values from 22 to 34 have previously been sequenced (John et al. 2021). Illumina COVIDSeq Test, Ion AmpliSeq SARS-CoV-2, and Oxford Nanopore are sequencing approaches predominantly used in animal diagnostics. John et al. provides a table comparing these three approaches and gives a LOD of detection as <500 copies/mL, 20 copies/reaction, and 10 copies**/**reaction, respectively (John et al. 2021). It is also important to highlight that each sequencing approach comes with unique parameters including, depth of coverage, length of read and single-end versus paired-end sequencing, and choice of approach is dependent on the sequencing goal and accessibility to technology. NGS is a critical aspect to SARS-CoV-2 testing and diagnostic, as knowing which variants are circulating amongst animal population will inform regulatory action affecting pets or wildlife.

NGS is an important part of clinical diagnostics. NGS can help identify mutations and biomarkers of novel or poorly characterized diseases. Importantly, sequencing assays are highly sensitive to detect SARS-CoV-2 even in samples that have low viral loads and sample preparation can be complete in little as 2.5 h. Illumina provides many different platforms that offer varying levels of throughput, including MiSeq, NextSeq, HiSeq and others. These Illumina approaches provide short read sequences that are assembled together to give a consensus sequence (Slatko et al. 2018). Additionally, long-full length reads can be accomplished through Oxford Nanopore’s MinION and other platforms, such as Grid- and PromethION, and may be advantageous, as it can easily identify mutation, although there is a relatively high error rate (Slatko et al. 2018). Illumina MiSeq and MinION are among the most common used sequencing approaches for SARS-CoV-2 diagnostics, as of the 4.8 million SARS-CoV-2 genomes deposited into GISAID (as of Nov. 2021), 65% used Illumina and 25% used Oxford Nanopore (Tshiabuila et al. 2022). A direct comparison of ONT GridION and Illumina MiSeq for SARS-CoV-2 sequencing, showed that the MiSeq had a significantly higher genome coverage, mutation counts, and overall number of consensus genomes (Tshiabuila et al. 2022).

Illumina sequencing has been used to confirm SARS-CoV-2 infection in many animal species, including, cats, tigers, deer, lions, and dogs (Leyi Wang et al. 2020; McAloose et al. 2020; Fernandez-Bellon et al. 2021; Klaus et al. 2021; Schiaffino et al. 2021; Zoccola et al. 2021; Hoppe et al. 2023; Kuchipudi et al. 2022; Lenz et al. 2022; Marques et al. 2022; Pickering et al. 2022) and MinION has been used for hamsters, opossums, tigers, armadillos, and dogs (McAloose et al. 2020; Arteaga et al. 2022; Chan et al. 2022; Goldberg et al. 2022; Rivero et al. 2022) (Table 3). Variants of interest are often discovered by NGS, as SARS-CoV-2 Lambda was first detected in domestic cats (Schiaffino et al. 2021). During the SARS-CoV-2 pandemic, sequencing animal samples has identified novel variants and transmission between species. An extensive study that included sequencing data from over 300 deer samples identified a highly divergent lineage of SARS-CoV-2 with 76 mutations and phylogenic evidence indicating a shared ancestry with mink-derived virus (Pickering et al. 2022). NGS has also illustrated the potential for variants to spill over in deer population from humans and then back to the human population (Kuchipudi et al. 2022; Pickering et al. 2022). Similar pattens were identified in mink, as SARS-CoV-2 was suggested to be transmittable back and forth between minks and mink farmers (Munnink et al. 2021). Other sequencing studies have directly proven transmission to companion animals from infected humans (Klaus et al. 2021; Zoccola et al. 2021; Hoppe et al. 2023; Rivero et al. 2022) and transmission from zookeepers to animals (McAloose et al. 2020). Sequencing from Virginian opossums showed new mutations in RBD, when aligned against the Wuhan variant, which were predicted to have increased binding affinity; these mutations were later sequenced in Omicron BJ.1 (Goldberg et al. 2022), indicating that infection in animals lead to novel mutation that may be more transmissible to humans. NGS diagnostics are critical to determining which variants are circulating in animal populations and assessing potential risk to human health.

**Table 3.** Sequencing platform used for SARS-CoV-2 animal testing.

Library preparation kit	Sequence platform	Reference	Species
Nextera XT Library Preparation Kit	Illumina NextSeq	(Lenz et al. 2022)	Cat
Native Barcode kit, EXP-NBD104, ligation sequencing kit, SQK-SQK109 (Oxford Nanopore Technologies)	Oxford Nanopore MinION	(McAloose et al. 2020)	Tiger and Lion
Nextera XT Library Preparation Kit	Illumina NextSeq	(Marques et al. 2022)	Deer
Nextera XT library prep kit	Illumina Hiseq 1500	(Hoppe et al. 2023)	Dog
Midnight RT-PCR protocol	Oxford Nanopore MinION	(Arteaga et al. 2022)	Armadillo
ARTIC nCoV-2019 protocol	Illumina NovaSeq 6000 instrument	(Kuchipudi et al. 2022)	Deer
EasySeq™ RC-PCR SARS-CoV-2 (novel coronavirus) Whole Genome Sequencing kit	Illumina MiSeq	(Zoccola et al. 2021)	Cat
NEBNext Ultra II DNA library	Illumina MiSeq	(Klaus et al. 2021)	Cat
Illumina DNA LibPrep kit	Illumina MiSeq	(Fernandez-Bellon et al. 2021)	Lion
Illumina COVIDSeq Test	Illumina MiniSeq	(Schiaffino et al. 2021)	Cat
Nextera DNA Flex Prep kit	Illumina MiniSeq	(Pickering et al. 2022)	Deer
Illumina Nextera XT DNA library	Illumina MiSeq Illumina iSeq	(Leyi Wang et al. 2020)	Tiger
Native Barcoding Expansion 96 (EXP-NBD196, Oxford Nanopore Technologies)	Oxford Nanopore MinION	(Chan et al. 2022)	Hamster
plexWell™ 384 Library Preparation Kit (seqWell, MA)	Illumina MiSeq	(Goldberg et al. 2022)	Opossum
ARTIC Network protocol	Oxford Nanopore MinION	(Rivero et al. 2022)	Dog

NGS has allowed for real-time tracking of emerging variants throughout the pandemic. In April 2020, D614G (pre-variant) was only detected in animals, but by early 2021 additional variants were found in animals, including Alpha, along with some detection of Epsilon, Iota, and Mu. By mid-2021 most of the detection was Delta, as this also became the dominate variant amongst human populations. Omicron first started to appear in animal populations in December 2021 and was carried throughout 2022 (USDA). Most of the animal cases were seen during the Delta spike in mid to end 2021 (Cui et al. 2022). Mutations, determined by NGS, in the Omicron variant have shown a reduction in the sensitivity in N-gene and S-gene targeting assays. Importantly, many of these tests are designed to target multiple genes, this way, a negative target along with two positive targets will not impact sensitivity and indicate a variant is present and should be sequenced (FDA n.db).

Compared to the Wuhan variant, Omicron has 37 mutations in the S gene leading to an increase in transmissibility and immune escape from vaccine-induced response in humans. It has been shown that companion animals are susceptible to the Omicron variant, however, when compared to the Delta variant, infection is relatively low. 40% of dogs and 31% cats (Meisner et al. 2021) become infected in households with a human infected with the Delta variant, whereas the infection rate with Omicron was relatively low with only 10% of animals becoming infected in homes with SARS-CoV-2 positive humans (Sanchez-Morales et al. 2022). A study directly compared the ratio of seropositive animals from SARS-CoV-2 infection homes during the peak infection of Delta and Omicron and found a reduced ratio from 50% −38.1% during Delta to 5.0% − 0.8% during Omicron (Klein et al. 2023). Similarly, infection with Omicron resulted in lower pathogenicity than Delta in felines (Martins et al. 2022). Interestingly, there is evidence that Omicron is more infectious in rodents than other animals. There has been an increase in infection in pet rodents (Montagutelli et al. 2022) and wild rodents. There is evidence that the Omicron has had a interspecies evolution. Hypothesized that it jumped from humans to mice, accumulated mutations, and then jumped back to humans.Omicron has three specific mutations that are also found in a mouse-adopted strain of SARS-CoV-2 which allows for more efficient binding to the mouse ACE2 receptor (Montagutelli et al. 2021; Wei et al. 2021; Montagutelli, Van Der Werf et al. 2022). There is less animal testing which in part accounts for a decrease in cases, however this may also be due to Omicron being less suited for animal hosts and more for humans. Understanding how spillovers from animal hosts living in SARS-CoV-2-infected households could speed the development of novel viral lineages and impact the accuracy and reliability of diagnostic tests is crucial. By knowing what variants are circulating in animal populations, diagnostic tests can be updated to ensure continued assay sensitivity.

## 6. Discussion

Cross-species transmission forces SARS-CoV-2 to adapt to new host environments resulting in species-specific mutations. Continued interspecies transmission allows for the potential for novel strain emergence. This has been demonstrated by the transmission of SARS-CoV-2 from human to mink and then back to humans (Munnink et al. 2021), as well as novel mutations being identified in both opossum and mice that were later seen in the Omicron variant (Montagutelli et al. 2021, Goldberg et al. 2022). Modifying animal diagnostic tests to better detect emerging variants will aid in active surveillance and genomic investigation. There are a range of tests that are used to determine active and past infection in animals. PCR is the most common, cost-effective, and sensitive assay. Limitations of PCR include sequence/variant detection, use is limited to the active phase of infection, and there is a lack of standardization between labs. Serology by ELISA and VNT are useful for diagnosing past infection which is critical to understanding transmission in a population or household. Determining sensitivity and specificity for an ELISA assay is important for accurate results. VNT require a BSL3 space and are variant-specific as the active virus used in the assay can affect neutralization. NGS is one of the most useful tools we have at determining variant of infection. It is also very sensitive, which is useful for samples with low viral amounts. Together, these various types of diagnostics for detection of SARS-CoV-2 each play an important role in understanding viral spread and impacts on animal and human health. Animals are engrossed in human lives, from everyday interaction with companion, farm, and zoo animals to the occasional encounter with wildlife, and zoonotic transmission of SARS-CoV-2 and other pathogens will continue to occur. Having multiple animal diagnostic tests available is critical to a One Health approach to ensure human and animal health.

## References

[CIT0001] Akhtardanesh B, Jajarmi M, Shojaee M, Tazerji SS, Mahani MK, Hajipour P, Gharieb R. 2023. Molecular screening of SARS-CoV-2 in dogs and cats from households with infected owners diagnosed with COVID-19 during Delta and Omicron variant waves in Iran. Vet Med Sci. 9(1):82–90. doi: 10.1002/vms3.1036. 36495219 PMC9856975

[CIT0002] Amicone M, Borges V, Alves MJ, Isidro J, Ze-Ze L, Duarte S, Vieira L, Guiomar R, Gomes JP, Gordo I. 2022. Mutation rate of SARS-CoV-2 and emergence of mutators during experimental evolution. Evol Med Public Health. 10(1):142–155. doi: 10.1093/emph/eoac010. 35419205 PMC8996265

[CIT0003] Arteaga FL, Jodar MN, Mondino M, Portu A, Boeris M, Joly A, Jar A, Mundo S, Castro E, Alvarez D, et al. 2022. An outbreak of SARS-CoV-2 in big hairy armadillos (*Chaetophractus villosus*) associated with Gamma variant in Argentina three months after being undetectable in humans. bioRxiv. doi: 10.1101/2022.08.23.503528.

[CIT0004] Barua S, Hoque M, Adekanmbi F, Kelly P, Jenkins-Moore M, Torchetti MK, Chenoweth K, Wood T, Wang CM. 2021. Antibodies to SARS-CoV-2 in dogs and cats, USA. Emerg Microbes Infect. 10(1):1669–1674. doi: 10.1080/22221751.2021.1967101. 34374631 PMC8381919

[CIT0005] Beattie RE, Blackwood AD, Clerkin T, Dinga C, Noble RT. 2022. Evaluating the impact of sample storage, handling, and technical ability on the decay and recovery of SARS-CoV-2 in wastewater. PLoS One. 17(6):e0270659. doi: 10.1371/journal.pone.0270659. 35749532 PMC9232146

[CIT0006] Bienzle D, Rousseau J, Marom D, MacNicol J, Jacobson L, Sparling S, Prystajecky N, Fraser E, Weese JS. 2022. Risk factors for SARS-CoV-2 infection and illness in cats and dogs. Emerg Infect Dis. 28(6):1154–1162. doi: 10.3201/eid2806.220423. 35608925 PMC9155877

[CIT0007] Bold D, Roman-Sosa G, Gaudreault NN, Zayat B, Pogranichniy RM, Richt JA. 2022. Development of an indirect ELISA for the detection of SARS-CoV-2 antibodies in cats. Front Vet Sci. 9:864884. doi: 10.3389/fvets.2022.864884. 35754530 PMC9226769

[CIT0008] Boszormenyi KP, Stammes MA, Fagrouch ZC, Kiemenyi-Kayere G, Niphuis H, Mortier D, van Driel N, Nieuwenhuis I, Vervenne RAW, Haaksma T, et al. 2021. The post-acute phase of SARS-CoV-2 infection in two macaque species is associated with signs of ongoing virus replication and pathology in pulmonary and extrapulmonary tissues. Viruses-Basel. 13(8):1673. doi: 10.3390/v13081673. PMC8402919 34452537

[CIT0009] Caceres PS, Savickas G, Murray SL, Umanath K, Uduman J, Yee J, Liao TD, Bolin S, Levin AM, Khan MN, et al. 2021. High SARS-CoV-2 viral load in urine sediment correlates with acute kidney injury and poor COVID-19 outcome. J Am Soc Nephrol. 32(10):2517–2528. doi: 10.1681/ASN.2021010059. 34088853 PMC8722807

[CIT0010] Carpenter A, Ghai RR, Gary J, Ritter JM, Carvallo FR, Diel DG, Martins M, Murphy J, Schroeder B, Brightbill K, et al. 2021. Determining the role of natural SARS-CoV-2 infection in the death of domestic pets: 10 cases (2020-2021). J Am Vet Med Assoc. 259(9):1032–1039. doi: 10.2460/javma.259.9.1032. 34647475 PMC10627034

[CIT0012] CDC. 2021. CDC 2019-novel coronavirus (2019-nCoV) real-time RT-PCR diagnostic panel. 10.1371/journal.pone.0260487 PMC8673615 34910739

[CIT0013] CDC. 2022. Interim guidelines for collecting and handling of clinical specimens for COVID-19 testing. https://www.cdc.gov/coronavirus/2019-ncov/lab/guidelines-clinical-specimens.html#handling-specimens-safely.

[CIT0014] Chan JFW, Siu GKH, Yuan SF, Ip JD, Cai JP, Chu AWH, Chan WM, Abdullah SMU, Luo CT, Chan BPC, et al. 2022. Probable animal-to-human transmission of severe acute respiratory syndrome coronavirus 2 (SARS-CoV-2) delta variant AY.127 causing a pet shop-related coronavirus disease 2019 (COVID-19) outbreak in Hong Kong. Clin Infect Dis. 75(1):E76–E81. doi: 10.1093/cid/ciac171. 35234870 PMC8903450

[CIT0015] Chandler JC, Bevins SN, Ellis JW, Linder TJ, Tell RM, Jenkins-Moore M, Root JJ, Lenoch JB, Robbe-Austerman S, DeLiberto TJ, et al. 2021. SARS-CoV-2 exposure in wild white-tailed deer (*Odocoileus virginianus*). Proc Natl Acad Sci U S A. 118(47): e2114828118. doi: 10.1073/pnas.2114828118. 34732584 PMC8617405

[CIT0016] Cui SJ, Liu YM, Zhao JC, Peng XM, Lu GL, Shi WX, Pan Y, Zhang DT, Yang P, Wang QY. 2022. An updated review on SARS-CoV-2 infection in animals. Viruses-Basel. 14(7):1527. doi: 10.3390/v14071527. PMC9323600 35891507

[CIT0017] Currie DW, Shah MM, Salvatore PP, Ford L, Whaley MJ, Meece J, Ivacic L, Thornburg NJ, Tamin A, Harcourt JL, et al. 2022. Relationship of SARS-CoV-2 antigen and reverse transcription PCR positivity for viral cultures. Emerg Infect Dis. 28(3):717–720. doi: 10.3201/eid2803.211747. PMC8888206 35202532

[CIT0018] Davoust B, Guerin P, Orain N, Fligny C, Flirden F, Fenollar F, Mediannikov O, Edouard S. 2022. Evidence of antibodies against SARS-CoV-2 in wild mustelids from Brittany (France). Transbound Emerg Dis. 69(5):E3400–E3407. doi: 10.1111/tbed.14663. 35841263 PMC9350122

[CIT0019] Decaro N, Grassi A, Lorusso E, Patterson EI, Lorusso A, Desario C, Anderson ER, Vasinioti V, Wastika CE, Hughes GL, et al. 2022. Long-term persistence of neutralizing SARS-CoV-2 antibodies in pets. Transbound Emerg Dis. 69(5):3073–3076. doi: 10.1111/tbed.14308. 34469620 PMC8662060

[CIT0020] Deng K, Nemser SM, Frost K, Goodman LB, Ip HS, Killian ML, Ulaszek J, Kiener S, Kmet M, Uhlig S, et al. 2022. Successful detection of delta and omicron variants of SARS-CoV-2 by veterinary diagnostic laboratory participants in an interlaboratory comparison exercise. J Appl Lab Med. 8(4):726–741. doi: 10.1093/jalm/jfad018. PMC11555767 37222567

[CIT0021] Deng K, Uhlig S, Goodman LB, Ip HS, Killian ML, Nemser SM, Ulaszek J, Kiener S, Kmet M, Frost K, et al. 2022. Second round of an interlaboratory comparison of SARS-CoV2 molecular detection assays used by 45 veterinary diagnostic laboratories in the United States. J Vet Diagn Invest. 34(5):825–834. doi: 10.1177/10406387221115702. 35983593 PMC9446291

[CIT0022] Deng K, Uhlig S, Ip HS, Lea Killian M, Goodman LB, Nemser S, Ulaszek J, Pickens S, Newkirk R, Kmet M, et al. 2021. Interlaboratory comparison of SARS-CoV2 molecular detection assays in use by U.S. veterinary diagnostic laboratories. J Vet Diagn Invest. 33(6):1039–1051. doi: 10.1177/10406387211029913. 34293974 PMC8532215

[CIT0023] Diao B, Wang CH, Wang RS, Feng ZQ, Zhang J, Yang H, Tan YJ, Wang HM, Wang CS, Liu L, et al. 2021. Human kidney is a target for novel severe acute respiratory syndrome coronavirus 2 infection. Nat Commun. 12(1):2506. doi: 10.1038/s41467-021-22781-1. 33947851 PMC8096808

[CIT0024] FAO. 2023, March 7. Food and agriculture Organization of the United Nations. “SARS-CoV-2 in animals situation update”. https://www.fao.org/animal-health/situation-updates/sars-cov-2-in-animals/en.

[CIT0025] FDA. n.da. In vitro diagnostics EUAs – Molecular diagnostic tests for SARS-CoV-2 | FDA. https://www.fda.gov/medical-devices/coronavirus-disease-2019-covid-19-emergency-use-authorizations-medical-devices/in-vitro-diagnostics-euas-molecular-diagnostic-tests-sars-cov-2#individual-molecular.

[CIT0026] FDA. n.db. SARS-CoV-2 viral mutations: impact on COVID-19 Tests. https://www.fda.gov/medical-devices/coronavirus-covid-19-and-medical-devices/sars-cov-2-viral-mutations-impact-covid-19-tests#other.

[CIT0027] Ferasin L, Fritz M, Ferasin H, Becquart P, Corbet S, Gouilh MA, Legros V, Leroy EM. 2021. Infection with SARS-CoV-2 variant B.1.1.7 detected in a group of dogs and cats with suspected myocarditis. Vet Rec. 189(9):e944. doi: 10.1002/vetr.944. 34738231 PMC8661638

[CIT0028] Fernandez-Bellon H, Rodon J, Fernandez-Bastit L, Almagro V, Padilla-Sole P, Lorca-Oro C, Valle R, Roca N, Grazioli S, Trogu T, et al. 2021. Monitoring natural SARS-CoV-2 infection in lions (*Panthera leo*) at the Barcelona Zoo: viral dynamics and host responses. Viruses-Basel. 13(9):1683. doi: 10.3390/v13091683. PMC8472846 34578266

[CIT0029] Fritz M, de Riols de Fonclare D, Garcia D, Beurlet S, Becquart P, Rosolen SG, Briend-Marchal A, Leroy EM. 2022. First evidence of natural SARS-CoV-2 infection in domestic rabbits. Vet Sci. 9(2):49. 35202302 10.3390/vetsci9020049 PMC8876202

[CIT0030] Gaudreault NN, Trujillo JD, Carossino M, Meekins DA, Morozov I, Madden DW, Indran SV, Bold D, Balaraman V, Kwon T, et al. 2020. SARS-CoV-2 infection, disease and transmission in domestic cats. Emerg Microbes Infect. 9(1):2322–2332. doi: 10.1080/22221751.2020.1833687. 33028154 PMC7594869

[CIT0031] Goldberg AR, Langwig KE, Marano J, Sharp AK, Brown KL, Ceci A, Kailing MJ, Briggs R, Roby C, Brown AM, et al. 2022. Wildlife exposure to SARS-CoV-2 across a human use gradient. bioRxiv. doi: 10.1101/2022.11.04.515237.

[CIT0032] Gontu A, Marlin EA, Ramasamy S, Neerukonda S, Anil G, Morgan J, Quraishi M, Chen C, Boorla VS, Nissly RH, et al. 2022. Development and validation of indirect enzyme-linked immunosorbent assays for detecting antibodies to SARS-CoV-2 in cattle, swine, and chicken. Viruses. 14(7):1358. doi: 10.3390/v14071358. 35891340 PMC9317974

[CIT0033] Hamer SA, Pauvolid-Corrêa A, Zecca IB, Davila E, Auckland LD, Roundy CM, Tang W, Torchetti MK, Killian ML, Jenkins-Moore M, et al. 2021. SARS-CoV-2 infections and viral isolations among serially tested cats and dogs in households with infected owners in Texas, USA. Viruses. 13(5):938. doi: 10.3390/v13050938. 34069453 PMC8159091

[CIT0034] Hayashi T, Abiko K, Mandai M, Yaegashi N, Konishi I. 2020. Highly conserved binding region of ACE2 as a receptor for SARS-CoV-2 between humans and mammals. Vet Q. 40(1):243–249. doi: 10.1080/01652176.2020.1823522. 32921279 PMC7580767

[CIT0035] Hoppe JM, Füeßl LU, Hartmann K, Hofmann-Lehmann R, Graf A, Krebs S, Blum H, Badell I, Keppler OT, Muenchhoff M. 2023. Secondary zoonotic dog-to-human transmission of SARS-CoV-2 suggested by timeline but refuted by viral genome sequencing. Infection. 51(1):253–259. doi: 10.1007/s15010-022-01902-y. 35986880 PMC9392066

[CIT0036] Huang X, Tang GY, Ismail N, Wang XW. 2022. Developing RT-LAMP assays for rapid diagnosis of SARS-CoV-2 in saliva. Ebiomedicine. 75:103736. doi: 10.1016/j.ebiom.2021.103736. 34922321 PMC8674011

[CIT0037] Jairak W, Chamsai E, Udom K, Charoenkul K, Chaiyawong S, Techakriengkrai N, Tangwangvivat R, Suwannakarn K, Amonsin A. 2022. SARS-CoV-2 delta variant infection in domestic dogs and cats, Thailand. Sci Rep. 12(1):8403. doi: 10.1038/s41598-022-12468-y. 35589808 PMC9117851

[CIT0038] John G, Sahajpal NS, Mondal AK, Ananth S, Williams C, Chaubey A, Rojiani AM, Kolhe R. 2021. Next-generation sequencing (NGS) in COVID-19: A tool for SARS-CoV-2 diagnosis, monitoring new strains and phylodynamic modeling in molecular epidemiology. Curr Issues Mol Biol. 43(2):845–867. doi: 10.3390/cimb43020061. 34449545 PMC8929009

[CIT0039] Kainulainen MH, Bergeron E, Chatterjee P, Chapman AP, Lee J, Chida A, Tang XL, Wharton RE, Mercer KB, Petway M, et al. 2021. High-throughput quantitation of SARS-CoV-2 antibodies in a single-dilution homogeneous assay. Sci Rep. 11(1):12330. doi: 10.1038/s41598-021-91300-5. 34112850 PMC8192771

[CIT0040] Klaus, J., M. L. Meli, B. Willi, S. Nadeau, C. Beisel, T. Stadler, V. S. T. Eth Sars-Co, H. Egberink, S. Zhao, H. Lutz, B. Riond, N. Rosinger, H. Stalder, S. Renzullo and R. Hofmann-Lehmann (2021). Detection and genome sequencing of SARS-CoV-2 in a domestic cat with respiratory signs in Switzerland. Viruses 13(3), 496. doi: 10.3390/v13030496. 33802899 PMC8002591

[CIT0041] Klaus J, Palizzotto C, Zini E, Meli ML, Leo C, Egberink H, Zhao S, Hofmann-Lehmann R. 2021. SARS-CoV-2 infection and antibody response in a symptomatic cat from Italy with intestinal B-cell lymphoma. Viruses. 13(3):527. doi: 10.3390/v13030527. 33806922 PMC8004793

[CIT0042] Klein C, Michelitsch A, Allendorf V, Conraths FJ, Beer M, Denzin N, Wernike K. 2023. Dogs and cats are less susceptible to the omicron variant of concern of SARS-CoV-2 – a field study. bioRxiv. doi: 10.1101/2023.02.07.527419. PMC12017219 40303725

[CIT0043] Kuchipudi SV, Surendran-Nair M, Ruden RM, Yon M, Nissly RH, Vandegrift KJ, Nelli RK, Li LL, Jayarao BM, Maranas CD, et al. 2022. Multiple spillovers from humans and onward transmission of SARS-CoV-2 in white-tailed deer. Proc Natl Acad Sci U S A. 119(6):e2121644119. doi: 10.1073/pnas.2121644119. 35078920 PMC8833191

[CIT0044] Laidoudi Y, Sereme Y, Medkour H, Watier-Grillot S, Scandola P, Ginesta J, Andreo V, Labarde C, Comtet L, Pourquier P, et al. 2021. SARS-CoV-2 antibodies seroprevalence in dogs from France using ELISA and an automated western blotting assay. One Health. 13:100293. doi: 10.1016/j.onehlt.2021.100293. 34377760 PMC8327341

[CIT0045] Lenz OC, Marques AD, Kelly BJ, Rodino KG, Cole SD, Perera RAPM, Weiss SR, Bushman FD, Lennon EM. 2022. SARS-CoV-2 delta variant (AY.3) in the feces of a domestic cat. Viruses-Basel. 14(2):421. doi: 10.3390/v14020421. PMC8877841 35216014

[CIT0046] Leyi Wang PKM, Calle PP, Bartlett SL, McAloose D, Killian ML, Yuan F, Fang Y, Goodman LB, Fredrickson R, Elvinger F, et al. 2020. Complete genome sequence of SARS-CoV-2 in a tiger from a U.S. Zoological Collection. Microbiol Resour Announc. 9(22);e00468–20. doi: 10.1128/MRA.00468-20. PMC7256270 32467283

[CIT0047] Mahdy MAA, Younis W, Ewaida Z. 2020. An overview of SARS-CoV-2 and animal infection. Front Vet Sci. 7:596391. doi: 10.3389/fvets.2020.596391. 33363234 PMC7759518

[CIT0048] Malta FD, Amgarten D, Val FC, Cervato MC, de Azevedo BMC, Basqueira MD, Alves COD, Nobrega MS, Reis RD, Sebe P, et al. 2021. Mass molecular testing for COVID19 using NGS-based technology and a highly scalable workflow. Sci Rep. 11(1):7122. doi: 10.1038/s41598-021-86498-3. 33782491 PMC8007582

[CIT0049] Mariano G, Farthing RJ, Lale-Farjat SLM, Bergeron JRC. 2020. Structural characterization of SARS-CoV-2: where we are, and where we need to be. Front Mol Biosci. 7:605236. doi: 10.3389/fmolb.2020.605236. 33392262 PMC7773825

[CIT0050] Marques AD, Sherrill-Mix S, Everett JK, Adhikari H, Reddy S, Ellis JC, Zeliff H, Greening SS, Cannuscio CC, Strelau KM, et al. 2022. Multiple introductions of SARS-CoV-2 alpha and delta variants into white-tailed deer in Pennsylvania. Mbio. 13(5):e0210122. doi: 10.1128/mbio.02101-22. 36000731 PMC9600874

[CIT0051] Martins M, Boggiatto PM, Buckley A, Cassmann ED, Falkenberg S, Caserta LC, Fernandes MHV, Kanipe C, Lager K, Palmer MV, et al. 2022. From deer-to-deer: SARS-CoV-2 is efficiently transmitted and presents broad tissue tropism and replication sites in white-tailed deer. PLoS Pathog. 18(3):e1010197. doi: 10.1371/journal.ppat.1010197. 35312736 PMC8970504

[CIT0052] Martins M, do Nascimento GM, Nooruzzaman M, Yuan FF, Chen C, Caserta LC, Miller AD, Whittaker GR, Fang Y, Diel DG. 2022. The omicron variant BA.1.1 presents a lower pathogenicity than B.1 D614G and delta variants in a feline model of SARS-CoV-2 infection. J Virol. 96(17):e0096122. doi: 10.1128/jvi.00961-22. 36000850 PMC9472624

[CIT0053] McAloose D, Laverack M, Wang LY, Killian ML, Caserta LC, Yuan FF, Mitchell PK, Queen K, Mauldin MR, Cronk BD, et al. 2020. From people to panthera: natural SARS-CoV-2 infection in tigers and lions at the Bronx Zoo. Mbio. 11(5):e02220–20. doi: 10.1128/mBio.02220-20. PMC7554670 33051368

[CIT0054] Meisner J, Baszler TV, Kuehl KH, Ramirez V, Baines A, Frisbie LA, Lofgren ET, Deavila DM, Wolking RM, Bradway DS, et al. 2021. Household transmission of SARS-CoV-2 from humans to pets in Washington and Idaho: burden and risk factors. Emerg Infect Dis. 28(12). 10.3201/eid2812.220215 PMC9707573 36288573

[CIT0055] Montagutelli X, Decaudin B, Beretta M, Mouquet H, Simon-Lorière E. 2022. SARS-CoV-2 infection in domestic rats after transmission from their infected owner. bioRxiv. doi: 10.1101/2022.10.13.512053.

[CIT0056] Montagutelli X, Prot M, Levillayer L, Salazar EB, Jouvion G, Conquet L, Beretta M, Donati F, Albert M, Gambaro F, et al. 2021. Variants with the N501Y mutation extend SARS-CoV-2 host range to mice, with contact transmission. bioRxiv. doi: 10.1101/2021.03.18.436013.

[CIT0057] Montagutelli X, Van Der Werf S, Rey FA, Simon-Loriere E. 2022. SARS-CoV-2 Omicron emergence urges for reinforced One-Health surveillance. EMBO Mol Med. 14(3):e15558. doi: 10.15252/emmm.202115558. 35083854 PMC8899904

[CIT0058] Morais O, Alves MR, Ramos C, Ferreira F, Fernandes P. 2022. The matrix effect in the RT-PCR detection of SARS-CoV-2 using saliva without rna extraction. Diagnostics. 12(7):1547. doi: 10.3390/diagnostics12071547. 35885453 PMC9318260

[CIT0059] Munnink BBO, Sikkema RS, Nieuwenhuijse DF, Molenaar RJ, Munger E, Molenkamp R, van der Spek A, Tolsma P, Rietveld A, Brouwer M, et al. 2021. Transmission of SARS-CoV-2 on mink farms between humans and mink and back to humans. Science. 371(6525):172–177. doi: 10.1126/science.abe5901. 33172935 PMC7857398

[CIT0060] Naqvi AA, Fatima K, Mohammad T, Fatima U, Singh IK, Singh A, Atif SM, Hariprasad G, Hasan GM, Hassan MI. 2020. Insights into SARS-CoV-2 genome, structure, evolution, pathogenesis and therapies: structural genomics approach. Biochim Biophys Acta-Mol Basis Disease. 1866(10):165878. 10.1016/j.bbadis.2020.165878 PMC7293463 32544429

[CIT0061] Pickering B, Lung O, Maguire F, Kruczkiewicz P, Kotwa JD, Buchanan T, Gagnier M, Guthrie JL, Jardine CM, Marchand-Austin A, et al. 2022. Divergent SARS-CoV-2 variant emerges in white-tailed deer with deer-to-human transmission. Nat Microbiol. 7(12):2011–2024. doi: 10.1038/s41564-022-01268-9. 36357713 PMC9712111

[CIT0062] Puelles VG, Lütgehetmann M, Lindenmeyer MT, Sperhake JP, Wong MN, Allweiss L, Chilla S, Heinemann A, Wanner N, Liu S, et al. 2020. Multiorgan and renal tropism of SARS-CoV-2. N Engl J Med. 383(6):590–592. doi: 10.1056/NEJMc2011400. 32402155 PMC7240771

[CIT0063] Rapichai W, Saejung W, Khumtong K, Boonkaewwan C, Tuanthap S, Lieberzeit PA, Choowongkomon K, Rattanasrisomporn J. 2022. Development of colorimetric reverse transcription loop-mediated isothermal amplification assay for detecting feline coronavirus. Animals. 12(16):2075. doi: 10.3390/ani12162075. 36009664 PMC9405184

[CIT0064] Ratti G, Lelli D, Moreno A, Stranieri A, Trogu T, Giordano A, Grassi A, Luzzago C, Decaro N, Paltrinieri S, et al. 2022. Comparison of diagnostic performances of different serological tests for SARS-CoV-2 antibody detection in cats and dogs. Transbound Emerg Dis. 69(6):3530–3539. doi: 10.1111/tbed.14716. 36183165 PMC9538080

[CIT0065] Rivero R, Garay E, Botero Y, Serrano-Coll H, Gastelbondo B, Munoz M, Ballesteros N, Castaneda S, Patino LH, Ramirez JD, et al. 2022. Human-to-dog transmission of SARS-CoV-2, Colombia. Sci Rep. 12(1):7880. doi: 10.1038/s41598-022-11847-9. 35551247 PMC9097567

[CIT0066] Roundy CM, Nunez CM, Thomas LF, Auckland LD, Tang W, Richison JJ, Green BR, Hilton CD, Cherry MJ, Pauvolid-Correa A, et al. 2022. High seroprevalence of SARS-CoV-2 in white-tailed deer (*Odocoileus virginianus*) at one of three captive cervid facilities in Texas. Microbiol Spectr. 10(2):e0057622. doi: 10.1128/spectrum.00576-22. 35319276 PMC9045306

[CIT0067] Rudd JM, Tamil Selvan M, Cowan S, Kao YF, Midkiff CC, Narayanan S, Ramachandran A, Ritchey JW, Miller CA. 2021. Clinical and histopathologic features of a feline SARS-CoV-2 infection model are analogous to acute COVID-19 in humans. Viruses. 13(8):1550. doi: 10.3390/v13081550. 34452415 PMC8402899

[CIT0068] Sanchez-Morales L, Sanchez-Vizcaino JM, Perez-Sancho M, Dominguez L, Barroso-Arevalo S. 2022. The OMICRON (B.1.1.529) SARS-CoV-2 variant of concern also affects companion animals. Front Vet Sci. 9:940710. doi: 10.3389/fvets.2022.940710. 36032286 PMC9411866

[CIT0069] Schiaffino F, Ferradas C, Jara LM, Salvatierra G, Davila-Barclay A, Sanchez-Carrion C, Ulloa A, Mascaro L, Pajuelo MJ, Sarmiento LG, et al. 2021. First detection and genome sequencing of SARS-CoV-2 Lambda (C.37) variant in symptomatic domestic cats in Lima, Peru. Front Vet Sci. 8:737350. doi: 10.3389/fvets.2021.737350. 34604373 PMC8484519

[CIT0070] Scialo F, Daniele A, Amato F, Pastore L, Matera MG, Cazzola M, Castaldo G, Bianco A. 2020. ACE2: the major cell entry receptor for SARS-CoV-2. Lung. 198(6):867–877. doi: 10.1007/s00408-020-00408-4. 33170317 PMC7653219

[CIT0071] Sethuraman N, Jeremiah SS, Ryo A. 2020. Interpreting diagnostic tests for SARS-CoV-2. Jama-Journal of the American Medical Association. 323(22):2249–2251. doi: 10.1001/jama.2020.8259. 32374370

[CIT0072] Sirakov I, Popova-Ilinkina R, Ivanova D, Rusenova N, Mladenov H, Mihova K, Mitov I. 2022. Development of nested PCR for SARS-CoV-2 detection and its application for diagnosis of active infection in cats. Vet Sci. 9(6):272. doi: 10.3390/vetsci9060272. 35737324 PMC9231004

[CIT0073] Slatko BE, Gardner AF, Ausubel FM. 2018. Overview of next-generation sequencing technologies. Curr Protoc Mol Biol. 122(1):e59. 29851291 10.1002/cpmb.59 PMC6020069

[CIT0074] Toohey-Kurth K, Crossley B, Hietala S. 2020. Focus issue on best practices for development, validation, and use of PCR assays. J Vet Diagn Invest. 32(6):757–757. doi: 10.1177/1040638720967122. 33140707 PMC7649540

[CIT0075] Tshiabuila D, Giandhari J, Pillay S, Ramphal U, Ramphal Y, Maharaj A, Anyaneji UJ, Naidoo Y, Tegally H, San EJ, et al. 2022. Comparison of SARS-CoV-2 sequencing using the ONT GridION and the Illumina MiSeq. BMC Genomics. 23(1):319. doi: 10.1186/s12864-022-08541-5. 35459088 PMC9026045

[CIT0076] USDA. 2023. Confirmed Cases of SARS-CoV-2 in Animals in the United States. https://www.aphis.usda.gov/aphis/dashboards/tableau/sars-dashboard.

[CIT0077] Vogels CBF, Brito AF, Wyllie AL, Fauver JR, Ott IM, Kalinich CC, Petrone ME, Casanovas-Massana A, Muenker MC, Moore AJ, et al. 2020. Analytical sensitivity and efficiency comparisons of SARS-CoV-2 RT-qPCR primer-probe sets. Nat Microbiol. 5(10):1299–1305. +. doi: 10.1038/s41564-020-0761-6. 32651556 PMC9241364

[CIT0078] Wei CS, Shan KJ, Wang WG, Zhang SY, Huan Q, Qian WF. 2021. Evidence for a mouse origin of the SARS-CoV-2 Omicron variant. J Genet Genomics. 48(12):1111–1121. doi: 10.1016/j.jgg.2021.12.003. 34954396 PMC8702434

[CIT0079] Wendling NM, Carpenter A, Liew A, Ghai RR, Gallardo-Romero N, Stoddard RA, Tao Y, Zhang J, Retchless AC, Ahmad A, et al. 2022. Transmission of SARS-CoV-2 Delta variant (B.1.617.2) from a fully vaccinated human to a canine in Georgia, July 2021. Zoonoses Public Health. 69(5):587–592. doi: 10.1111/zph.12944. 35426241 PMC9115446

[CIT0080] Wernike K, Aebischer A, Michelitsch A, Hoffmann D, Freuling C, Balkema-Buschmann A, Graaf A, Müller T, Osterrieder N, Rissmann M, et al. 2021. Multi-species ELISA for the detection of antibodies against SARS-CoV-2 in animals. Transbound Emerg Dis. 68(4):1779–1785. doi: 10.1111/tbed.13926. 33191578 PMC7753575

[CIT0081] Zhang Q, Zhang HJ, Gao JD, Huang K, Yang Y, Hui XF, He XL, Li CF, Gong WX, Zhang YF, et al. 2020. A serological survey of SARS-CoV-2 in cat in Wuhan. Emerg Microbes Infect. 9(1):2013–2019. doi: 10.1080/22221751.2020.1817796. 32867625 PMC7534315

[CIT0082] Zhang Y, Huang Z, Zhu J, Li C, Fang Z, Chen K, Zhang Y. 2022. An updated review of SARS-CoV-2 detection methods in the context of a novel coronavirus pandemic. Bioeng Transl Med. 8(1):e10356. doi: 10.1002/btm2.10356. 35942232 PMC9349698

[CIT0083] Zoccola R, Beltramo C, Magris G, Peletto S, Acutis P, Bozzetta E, Radovic S, Zappulla F, Porzio AM, Gennero MS, et al. 2021. First detection of an Italian human-to-cat outbreak of SARS-CoV-2 Alpha variant-lineage B.1.1.7. One Health. 13:100295. doi: 10.1016/j.onehlt.2021.100295. 34316508 PMC8299139

